# Effect of levodopa-carbidopa intestinal gel on resting tremors in patients with advanced Parkinson’s disease

**DOI:** 10.1038/npjparkd.2016.15

**Published:** 2016-07-14

**Authors:** Hubert H Fernandez, Weining Z Robieson, Krai Chatamra, Jordan Dubow, Susan Eaton, Janet A Benesh, Per Odin

**Affiliations:** 1 Center for Neurological Restoration, Cleveland Clinic, Cleveland, OH, USA; 2 AbbVie Inc., North Chicago, IL, USA; 3 Klinikum Bermerhaven, Bremerhaven, Germany and Skane University Hospital, Lund, Sweden

## Abstract

Resting tremors occur in more than 70% of patients with advanced Parkinson’s disease (PD). PD patients with resting tremors are typically treated with oral dopaminergic therapy or non-dopaminergic agents. However, treatment response with these medications is inconsistent and often unsatisfactory. Levodopa-carbidopa intestinal gel (LCIG, also known in the United States as carbidopa-levodopa enteral suspension (CLES)), administered continuously by a portable pump via a percutaneous endoscopic gastrojejunostomy (PEG-J) tube, significantly improves motor complications in patients with advanced PD. This was a *post hoc* analysis of a large phase 3, 12-month, open-label study evaluating long-term safety and efficacy of LCIG via PEG-J tube (NCT00335153). Unified Parkinson’s Disease Rating Scale Part III Question 20 total scores at baseline, measuring resting tremors, were used to stratify patients into three subgroups (none, mild, or significant baseline resting tremors). Out of 354 enrolled patients, 286 had baseline and post-PEG-J assessments of resting tremors and were included in this analysis. At baseline the majority of patients (69%) had no resting tremors, whereas 13% had mild resting tremors, and 18% had significant resting tremors. A complete resolution in resting tremors after 12 months of LCIG treatment was reported for 78% and 70% of patients with mild and significant baseline resting tremors, respectively. Improvements in motor complications and quality of life occurred regardless of degree of baseline resting tremors. LCIG may provide more consistent and sustained improvements in resting tremors that were not well-controlled with optimized oral medication among patients with advanced PD.

## Introduction

Resting tremors are common in patients with advanced Parkinson’s disease (PD), with more than 70% of patients reporting resting tremors during the course of their disease.^1^ Some PD patients also report postural and action tremors that occur during voluntary movement^2^ along with these resting tremors.^3^ Dopaminergic therapies (e.g., levodopa, pramipexole) or non-dopaminergic agents (e.g., amantadine and anticholinergic agents) are typically used to treat resting tremors and other symptoms associated with PD.^2^ However, levodopa treatment is commonly associated with motor fluctuations, which are caused, in part, by the short half-life of levodopa and irregular gastric emptying resulting in inconsistent plasma levodopa concentrations.^4,5^ Other oral agents may cause additional idiosyncratic or class side effects. More importantly, resting tremors are not consistently controlled with available oral therapies and are one of the main indications for invasive therapeutic procedures such as deep brain stimulation.^2,6,7^

Levodopa-carbidopa intestinal gel (LCIG, also known in the United States as carbidopa-levodopa enteral suspension (CLES)) is administered by a portable pump via a percutaneous endoscopic gastrojejunostomy (PEG-J) tube and delivers continuous infusion of levodopa therapy to decrease motor fluctuations.^5^ LCIG administration bypasses the stomach, thus eliminating issues that may arise from variable gastric emptying.^4^ LCIG treatment via PEG-J significantly reduces ‘Off’ time and significantly increases ‘On’ time without troublesome dyskinesia (TSD).^5,8,9^

The effect of LCIG and continuous levodopa treatment on resting tremors has not been investigated to date. Therefore, we assessed the effect of LCIG on resting tremors in a large, multinational, open-label study of patients with advanced PD.

## Results

### Patients

Out of the 354 patients enrolled in the study, 286 had both baseline and post-PEG-J assessments of UPDRS Part III Question 20 (resting tremors) and were included in this analysis (Table 1). At baseline, the majority of patients (69%) had no resting tremor, whereas 13% had mild resting tremors, and 18% had significant resting tremors. Patients had similar mean ‘off’ and ‘on’ times and similar oral levodopa and amantadine doses at baseline across all three baseline resting tremor subgroups. At baseline, more patients had some level of action tremors (138/286 (48%)) than some level of resting tremors (90/286 (31%)); however, significant resting tremors was more common (*n*=52) than significant action tremors (UPDRS Part III Question 21 maximum score ⩾2, *n*=36). At baseline, the mean (s.d.) sleep time was similar between the subgroups (no baseline resting tremor=7.20 (1.79) hours; mild baseline resting tremor=7.46 (1.80) hours; significant baseline resting tremor=7.43 (2.30) hours).

After 12 months of LCIG treatment, the mean increase in total daily levodopa dose from baseline to final was similar in all three subgroups, with a mean (s.d.) change from baseline to final levodopa dose of 423.0 (646.6) mg for the no baseline resting tremor subgroup, 336.4 (585.4) mg for the mild baseline resting tremor subgroup, and 441.0 (546.0) mg for the significant baseline resting tremor subgroup. Amantadine use decreased across all three subgroups from ~30% use at baseline (Table 1) to 10, 8, and 12% use for the no baseline resting tremor, mild baseline resting tremor, and significant baseline resting tremor subgroups, respectively.

#### Efficacy assessments

**Figure 1:**
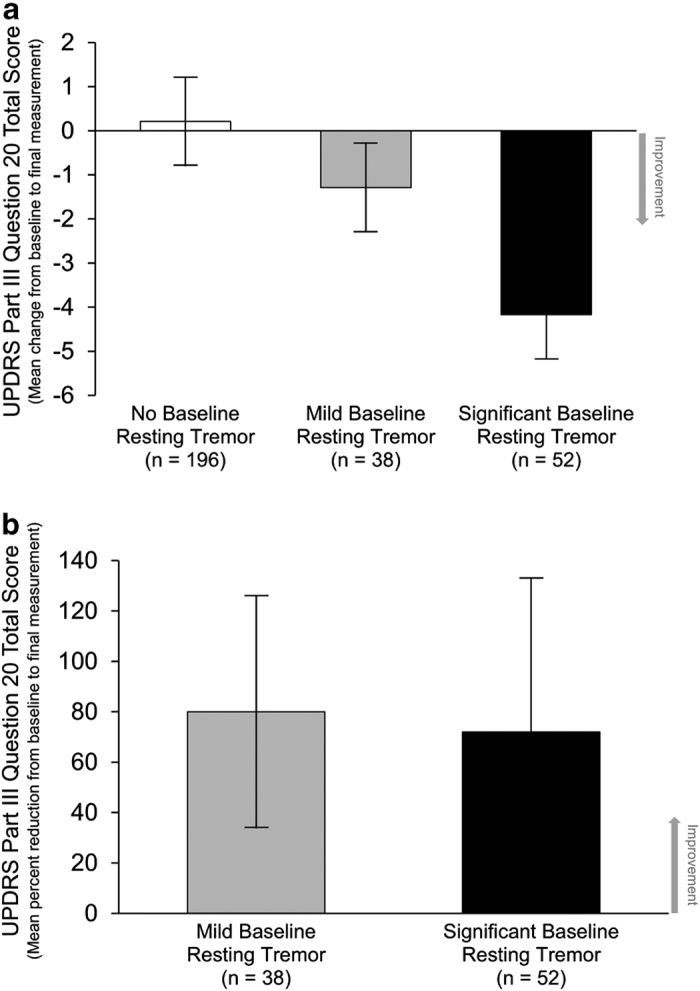
Change in resting tremors (UPDRS Part III Question 20 total score). UPDRS Part III Question 20 total score (**a**) mean change from baseline to final measurement and (**b**) mean percent reduction from baseline to final measurement in groups with baseline resting tremor (mild and significant baseline resting tremors). Data are the mean±s.d. UPDRS, Unified Parkinson’s Disease Rating Scale.

The mean change from baseline in UPDRS Part III Question 20 total score was greater in patients with significant baseline resting tremors than in patients with mild baseline resting tremors (Figure 1a). Because of their higher baseline resting tremor scores, patients with significant baseline resting tremors had the potential to experience a greater reduction (i.e., improvement) in score. However the percent reduction in resting tremor scores from baseline was similar in the two patient subgroups with baseline resting tremors; the mild baseline resting tremor subgroup had an 80% reduction in tremors and the significant baseline resting tremor subgroup experienced a 72% reduction in tremors (Figure 1b). Moreover, patients with either mild or significant baseline resting tremors experienced reduction in tremors concurrent with a decrease in the use of amantadine.

**Figure 2:**
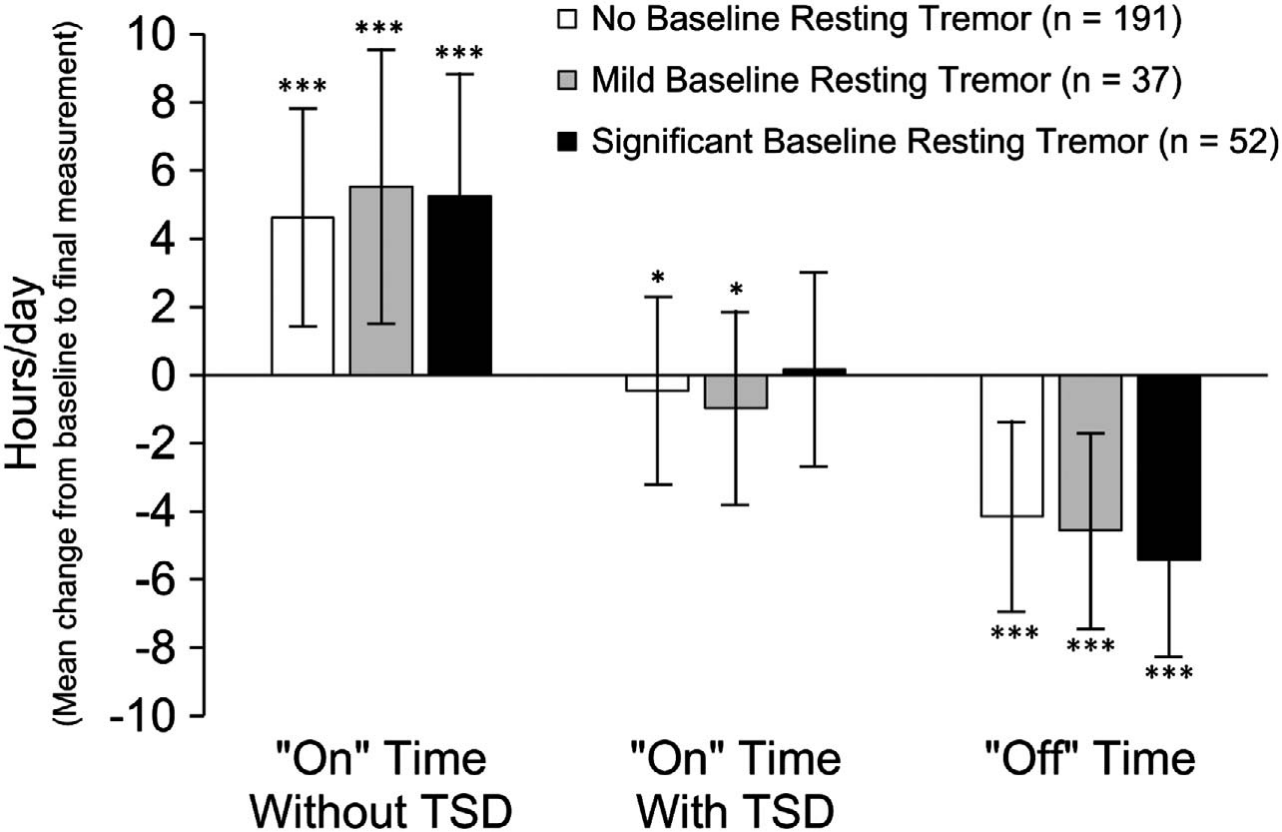
Mean change from baseline in ‘Off’ time, ‘On’ time without TSD, and ‘On’ time with TSD. Data are the mean±s.d. Statistical significance versus baseline was calculated with a one-sample *t-*test, indicated by *P<0.05 and ***P<0.001. TSD, troublesome dyskinesia.

After 12 months of LCIG treatment, there was a significant (*P*<0.001) improvement from baseline in ‘Off’ time and ‘On’ time without TSD for all three baseline resting tremor subgroups (Figure 2). In addition, there was a significant (*P*<0.05) reduction from baseline in ‘On’ time with TSD for the no baseline resting tremor subgroup and the mild baseline resting tremor subgroup.

**Figure 3:**
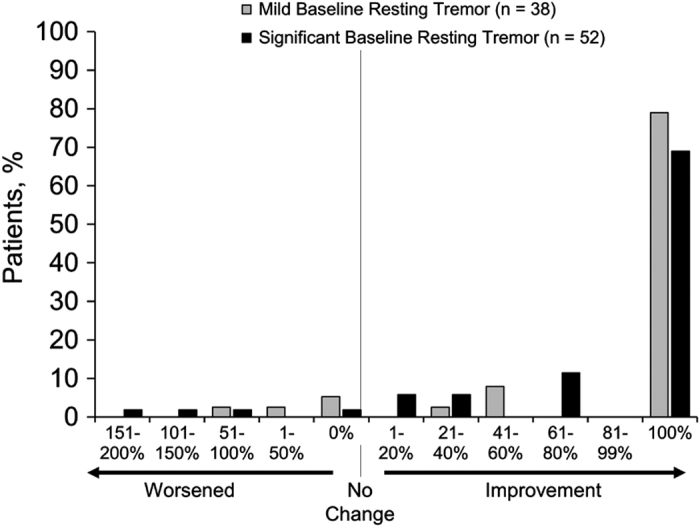
Percent of change from baseline to final measurement in UPDRS Part III Question 20 total score. UPDRS, Unified Parkinson’s Disease Rating Scale.

A majority of patients (78% and 70% with mild and significant baseline resting tremors, respectively) experienced a complete resolution in resting tremors after 12 months of treatment with LCIG (Figure 3). Just two patients (5%) in the mild baseline resting tremor subgroup and three patients (6%) in the significant baseline resting tremor subgroup experienced an increase in maximum resting tremor score.

**Figure 4:**
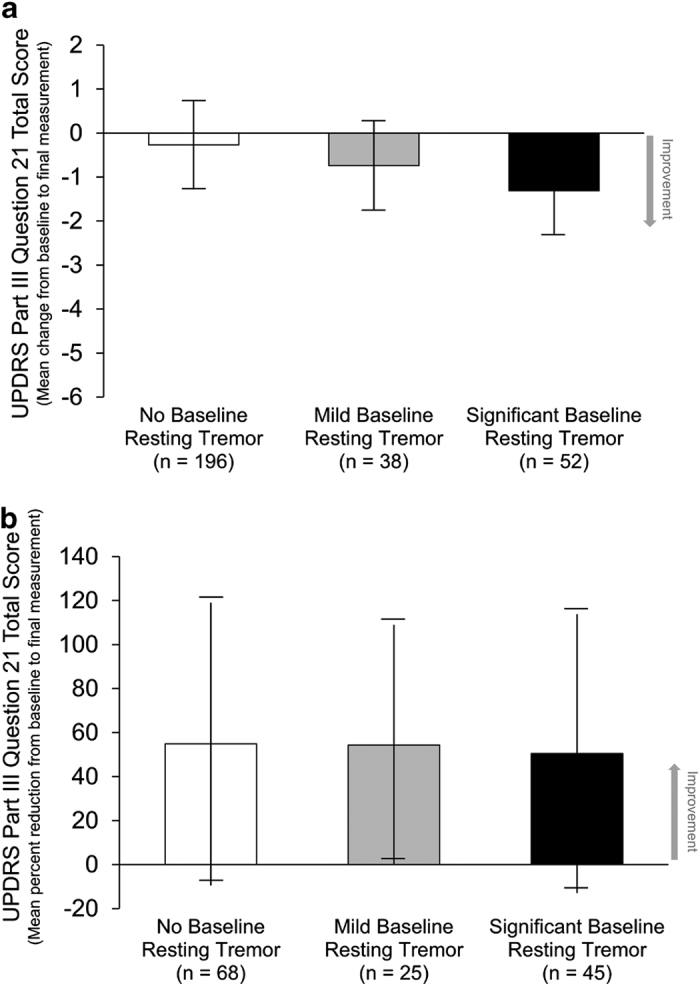
Change in action tremors (UPDRS Part III Question 21 total score). UPDRS Part III Question 21 total score (**a**) mean change from baseline to final measurement and (**b**) mean percent reduction from baseline to final measurement in patients with baseline action tremors. Data are the mean±s.d. UPDRS, Unified Parkinson’s Disease Rating Scale.

The significant baseline resting tremor group had a higher mean (s.d.) baseline action tremor total score (2.37 (1.77)) compared with total scores in the mild (1.29 (1.16)) and no baseline (0.64 (1.08)) resting tremor subgroups (Table 1). However, the percent decrease in action tremor total scores were similar across the baseline resting tremor subgroups (Figure 4).

Patients in the three baseline resting tremor subgroups experienced an improvement in quality of life from baseline to final visit as demonstrated by PDQ-39 Summary Index. The improvements in patients across the three baseline resting tremor subgroups were similar, with a mean (s.d.) change from baseline of −6.91 (13.78), −8.14 (13.34), and −8.03 (15.92) for patients with no, mild, or significant baseline resting tremors, respectively. Changes in sleep between groups were comparable, with a mean (s.d.) change from baseline of 0.44 (2.04) hour, −0.14 (1.98) hour, and 0.58 (2.10) hour for patients with no, mild, or significant baseline resting tremors, respectively.

#### Safety assessments

Adverse events (AEs) were reported in 94% of enrolled patients and serious AEs (SAEs) were reported in 32% of patients during the study.^8^ AE and SAE incidences were similar across the three baseline resting tremor subgroups (Table 2). The most commonly reported SAE was complication of device insertion, occurring in 5%, 8%, and 4% of patients with no baseline resting tremor, mild baseline resting tremors, and significant baseline resting tremors, respectively. Tremors were reported as an AE in four patients (2%) in the no baseline resting tremor subgroup, one patient (3%) in the mild baseline resting tremor subgroup, and one patient (2%) in the significant baseline resting tremor subgroup. Tremors were not reported as an SAE in any of the baseline resting tremor subgroups.

## Discussion

Resting tremors are one of the most common and perhaps the most visible of all symptoms of PD, and have a negative impact on the quality of life of patients with the disease. Patients with PD tremors report difficulties with many daily activities, such as ability to write/type, use a phone, fix small things, and get dressed.^10^ PD tremors are also associated with non-motor symptoms, such as negative feelings, loneliness, embarrassment, anxiety, and depression, all of which contribute further to a reduced quality of life.^10,11^

PD resting tremors are not often the focus of treatment. Pharmacologic management of PD resting tremors includes oral levodopa, dopamine agonists, monoamine oxidase B inhibitors, catechol-*o*-methyltransferase inhibitors, anticholinergic drugs, and amantadine; however, PD resting tremors are not always responsive to these treatments.^2^ Alternative pharmacological therapies, particularly if resting tremors are associated with action and postural tremors, include anticonvulsants (e.g., primidone, gabapentin), β-blocking agents, NMDA receptor antagonists (e.g., acamprosate), and atypical antipsychotics (e.g., clozapine).^12^ Other therapies have been developed to address the lack of responsiveness to pharmacologic treatment of PD resting tremors,^13^ including botulinum toxin type A injection.^14^ However, resting tremor localization between patients varies, therefore, using botulinum toxin type A injections to treat PD resting tremors is problematic. Deep brain stimulation is a more established therapy used to treat oral PD medication-refractory tremors; nevertheless, the invasiveness, expense, and potential side effects that may develop from this therapy limit its widespread use for this particular indication. Moreover, findings from studies of deep brain stimulation have shown that the surgical procedure^15,16^ and electrode placement^17^ may negatively affect cognition and verbal fluency.^15,16^

We investigated whether treatment of PD with LCIG could alleviate resting tremors in patients with advanced PD that was not well-controlled by optimized oral medication. In this *post hoc* analysis, 80% and 72% of patients with mild and significant baseline resting tremors, respectively, experienced a reduction in tremors. Moreover, a majority of patients with resting tremors at baseline experienced a complete resolution in resting tremors after 12 months of treatment with LCIG. Improvements in ‘Off’ time and ‘On’ time without TSD in patients treated with LCIG occurred independent of the degree of baseline resting tremors. In each baseline resting tremor subgroup, action tremors decreased from baseline by approximately 50%. Patient quality of life, as assessed by PDQ-39 Summary Index, improved regardless of baseline resting tremor subgroup. Changes in mean sleep time were comparable between subgroups. SAEs were similar among the three baseline resting tremor subgroups, and were also similar to reports made in the overall study.^8^

The mechanism by which continuous levodopa infusion may further improve resting tremors in PD patients, compared with that of oral dopaminergic therapy, is unclear. Observed improvements in tremors with LCIG treatment could be because levodopa/carbidopa is dosed continuously, resulting in reduced plasma levodopa fluctuations when compared with oral dosing, thus allowing patients to achieve the more effective dose needed to control resting tremors. Improved tolerability (e.g., fewer dyskinesias) seen with continuously dosed levodopa allows for larger doses of levodopa, if necessary, when compared with oral therapy;^5,8,9^ the overall higher dose of levodopa/carbidopa administered to patients in this study compared with typical oral dosing may have improved control of resting tremors. However, the dose of levodopa/carbidopa increased to a similar degree in the mild and significant resting tremor subgroups, yet there was a larger improvement in resting tremor in the significant baseline tremor subgroup. It is also possible that with less ‘Off’ time produced by continuous LCIG administration, fewer tremors are occurring. This overall reduction in tremors with LCIG treatment is demonstrated by the similar percent reduction in action tremors seen in all three baseline resting tremor subgroups.

This study had a population bias that excluded patients with predominant medication-resistant tremors, which could have affected the results. A majority of patients in this analysis had no baseline resting tremors, which may be because patients’ resting tremors were already controlled by pre-study PD medication. Although the Unified Parkinson’s Disease Rating Scale (UPDRS) Part III Question 20 is a recognized, reliable scale used to assess tremors, completion of Part III Question 20 relies on the investigator’s perception of the patients’ tremors only at the time of scoring. In particular, UPDRS was assessed at patients’ best ‘On’ time, which could reflect a lower overall tremor status than what patients may experience throughout the day as their motor symptoms fluctuate between ‘On’ and ‘Off’ times. In addition, it has been reported that there are diurnal fluctuations in tremors,^18^ and that tremors are affected by sleep.^19^ The patients in this study had comparable baseline sleep times as well as change in sleep times from baseline. However, variations between patients in the time of UPDRS Part III Question 20 scoring could affect how patients were scored and separated into baseline resting tremor subgroups. As an alternative to investigator-scored UPDRS Part III Question 20, Kostikis *et al*.^20^ have demonstrated the use of a smartphone-based tool that can measure tremors directly, allowing for more frequent monitoring of PD tremors during a study. Due to the *post hoc* nature of the analysis reported herein, and the open-label treatment study design, the effect of LCIG on reducing resting tremors in this population of patients with advanced PD needs to be prospectively investigated. Nonetheless, results from this analysis demonstrated that LCIG may further improve tremors, regardless of baseline resting tremor intensity; improve resting tremors that are not well-controlled by oral levodopa; and, perhaps in select patients, provide an alternative to more invasive procedures, such as deep brain stimulation, for the treatment of resting tremors.

## Materials and Methods

### Study design and treatment

This was a *post hoc* analysis of a large, multinational, phase 3, 12-month, open-label study that evaluated long-term safety and efficacy of LCIG administered via PEG-J tube (NCT00335153).^8^ The study protocol was approved by the institutional review board/ethics committee at all 86 centers in 16 countries and was conducted in accordance with the principles of the Declaration of Helsinki, good clinical practice, and applicable regulatory requirements. All patients provided written informed consent.^8^

The study included a screening period (up to 28 days), a nasojejunal (NJ) titration period (2–14 days), a PEG-J titration period (2–14 days), and a 54-week treatment period. Patients were tapered off any PD medication and stabilized on oral carbidopa-levodopa monotherapy, before undergoing the NJ titration period to test the clinical response to LCIG treatment. Patients then underwent surgery for PEG-J tube placement; subsequently, LCIG was administered continuously via a portable pump during 16 h of wakefulness.

### Patients

Patients eligible for this study were ⩾30 years of age, had a diagnosis of idiopathic PD according to the United Kingdom Parkinson’s Disease Society Brain Bank criteria, were levodopa-responsive as observed by the investigator, and had severe motor fluctuations defined as ⩾3 h of daily ‘Off’ time at baseline despite individually optimized pharmacological therapy.^8^

### Assessments

#### Efficacy assessments

Efficacy outcomes included the mean change from baseline to the last visit in ‘Off’ time, ‘On’ time without TSD (i.e., does not interfere with daily functions or cause any significant discomfort), and ‘On’ time with TSD, all of which were recorded by patients in the PD symptom diary.^8^ The UPDRS was administered to each patient by the investigator during the patient’s best ‘On’ state. In addition, the 39-item PD Questionnaire (PDQ-39) was used to assess patients’ quality of life. Diary measures (including a sleep log) and UPDRS and PDQ-39 scores were assessed at weeks 4, 12, 24, and 54 after PEG-J placement; diary measures were also collected at week 36.^8^

The focus of this *post hoc* analysis is UPDRS Part III Question 20, which measures resting tremors based on five categories (that include the head and upper and lower extremities), and UPDRS Part III Question 21, which measures action or posture tremors of each hand.

#### Safety assessment

Treatment-emergent AEs were monitored throughout the study, coded using the Medical Dictionary for Regulatory Activities (MedDRA) version 14.0, and tabulated by MedDRA system organ class and preferred term. AEs could be coded to more than one preferred term. For example, AEs that were coded to the preferred term ‘complication of device insertion’ could also be coded to one or more terms descriptive of the event presentation such as ‘abdominal pain’, ‘abdominal discomfort’, or ‘abdominal distention’.^8^

### Statistical analysis

The maximum score from the five categories of UPDRS Part III Question 20 (resting tremors) at baseline was used to stratify patients into three subgroups. These subgroups included patients with no baseline resting tremor (score of 0 in all body parts), mild baseline resting tremors (maximum score of 1 in any body part), or significant baseline resting tremors (maximum score of ⩾2 in any body part). Efficacy and safety were evaluated in this *post hoc* analysis in the context of these three baseline resting tremor subgroups. UPDRS Part III Question 20 total score for each visit was calculated as the sum of the scores from the five categories (range, 0–20). UPDRS Part III Question 21 total score for each visit was calculated as the sum of the scores from the two categories (range, 0–8). PD symptom diary measures were normalized to a 16-hour waking day and averaged over the 3 days before each visit. The mean change from baseline to final visit in all efficacy variables was analyzed using a one-sample *t*-test. Descriptive statistics were used to evaluate the differences in demographics and safety across baseline resting tremor subgroups.

## Figures and Tables

**Table 1:** Baseline demographics and disease characteristics

*Characteristic*	*No baseline resting tremor* n*=196*	*Mild baseline resting tremor* n*=38*	*Significant baseline resting tremor* n*=52*
Mean age, years (s.d.)	64.7 (9.2)	60.7 (8.5)	64.1 (9.7)
			
*Sex,* n *(%)*			
Male	112 (57)	26 (68)	31 (60)
Female	84 (43)	12 (32)	21 (40)
Mean PD duration, years (s.d.)	12.1 (4.7)	11.6 (6.2)	13.0 (7.4)
Range	2.8–23.2	1.5–34.7	3.4–31.3
Mean levodopa dose at baseline, mg/day (s.d.)	1115 (589)^a^	1161 (674)	1021 (478)^b^
Amantadine use at baseline, n (%)	61 (31)	12 (32)	14 (27)
‘Off’ time, hours/day, mean (s.d.)	6.62 (2.18)	6.88 (2.08)	7.34 (2.98)
‘On’ time with TSD, hours/day, mean (s.d.)	1.67 (2.14)	1.88 (2.00)	1.43 (1.78)
‘On’ time without TSD, hours/day, mean (s.d.)	7.71 (2.29)	7.24 (2.72)	7.23 (2.60)
UPDRS Part III Question 20 (resting tremors) total score^c^, mean (s.d.)	0 (0)	1.69 (0.8)	5.25 (2.6)
UPDRS Part III Question 21 (action tremors) total score^d^, mean (s.d.)	0.64 (1.08)	1.29 (1.16)	2.37 (1.77)
PDQ-39 SI, mean (s.d)	42.81 (14.86)	45.30 (13.20)	43.16 (18.02)

Abbreviations: PD, Parkinson’s disease; PDQ-39 SI, PD Questionnaire 39 summary index; TSD, troublesome dyskinesia; UPDRS, Unified Parkinson’s Disease Rating Scale.

a *n*=192.

b *n*=51.

c Sum of UPDRS Part III Question 20 scores from the five categories (head, upper and lower extremities), with a possible range of 0–20.

d Sum of UPDRS Part III Question 21 scores from the two categories (left and right hands), with a possible range of 0–8.

**Table 2:** Incidence of serious treatment-emergent adverse events occurring in ⩾2% of patients^a^

	*Patients,* n *(%)*		
	*No baseline resting tremor* n*=196*	*Mild baseline resting tremor* n*=38*	*Significant baseline resting tremor* n*=52*
Any serious adverse event	62 (32)	9 (24)	21 (40)
Complication of device insertion	9 (5)	3 (8)	2 (4)
Polyneuropathy	6 (3)	1 (3)	1 (2)
Parkinson’s disease	4 (2)	1 (3)	3 (6)
Pneumoperitoneum	3 (2)	1 (3)	2 (4)
Weight decreased	1 (1)	0	2 (4)
Prostate cancer	0	0	2 (4)
Psychotic disorder	0	0	2 (4)

a Adverse events could be coded to >1 preferred term. Preferred terms listed in descending order of patient incidence for the no baseline tremor subgroup.
